# TRIM25, TRIM28 and TRIM59 and Their Protein Partners in Cancer Signaling Crosstalk: Potential Novel Therapeutic Targets for Cancer

**DOI:** 10.3390/cimb46100638

**Published:** 2024-09-25

**Authors:** De Chen Chiang, Beow Keat Yap

**Affiliations:** School of Pharmaceutical Sciences, Universiti Sains Malaysia, Gelugor, Penang 11800, Malaysia

**Keywords:** tripartite motif, cancer, pathway, TRIM25, TRIM28, TRIM59

## Abstract

Aberrant expression of TRIM proteins has been correlated with poor prognosis and metastasis in many cancers, with many TRIM proteins acting as key oncogenic factors. TRIM proteins are actively involved in many cancer signaling pathways, such as p53, Akt, NF-κB, MAPK, TGFβ, JAK/STAT, AMPK and Wnt/β-catenin. Therefore, this review attempts to summarize how three of the most studied TRIMs in recent years (i.e., TRIM25, TRIM28 and TRIM59) are involved directly and indirectly in the crosstalk between the signaling pathways. A brief overview of the key signaling pathways involved and their general cross talking is discussed. In addition, the direct interacting protein partners of these TRIM proteins are also highlighted in this review to give a picture of the potential protein–protein interaction that can be targeted for future discovery and for the development of novel therapeutics against cancer. This includes some examples of protein partners which have been proposed to be master switches to various cancer signaling pathways.

## 1. Introduction

Tripartite motif (TRIM) proteins represent a family of proteins with unique and crucial roles in modulating innumerable cellular processes, including cell cycle regulation, apoptosis, antiviral response, cellular development and carcinogenesis [1,2,3,4]. TRIM family proteins are also known as the RING, B-box and coiled-coil (RBCC) family proteins, as they generally have a conserved protein architecture characterized by three zinc-binding domains, i.e., a RING domain (R), one or two B boxes (B1 and B2) and a coiled-coil region (CC) at their N-terminus [2,5]. At the other end, TRIM family proteins contain 10 different types of C-terminal domains, such as the PRY and SPRY domains, which allow TRIM proteins to be further categorized into 11 subgroups [3].

In general, there are more than 80 TRIM protein genes identified in humans, in which most TRIM proteins are defined as E3 ubiquitin ligases due to the ubiquitination ability of their N-terminal RING domain [1]. Ubiquitin-mediated proteasomal degradation effectively degrades proteins through a cascade process, in which ubiquitin is conjugated to the target protein followed by proteasome-mediated degradation [6]. Interaction among the CC domains of different TRIM proteins forms polymers, structurally regulating E3 ligase substrate recognition [7]. On the other hand, the C-terminus of TRIM proteins harbors the substrate-binding domain, giving rise to their structural diversity for a vast range of substrate interactions in immune signaling, pathogen recognition and protein–protein interactions [4,8,9,10].

Extensive studies have demonstrated that the aberrant expression of TRIM proteins correlates with poor prognosis and metastasis in many cancers, in which many TRIM proteins act as the key oncogenic factor [1,11]. Most TRIM proteins, such as TRIM44 and TRIM59, exhibit a cancer-promoting role, consistently upregulated across several cancer types [12,13,14,15,16,17,18,19,20,21,22,23,24,25,26], whereas TRIM proteins like TRIM16 suppress cell proliferation and metastasis in neuroblastoma, melanoma, ovarian cancer and breast cancer [27,28,29,30]. On the flip side, some TRIM proteins demonstrate both cancer-causing and anti-cancer effects across different malignancies. For example, TRIM29 expression is upregulated in head and neck squamous cell carcinoma, nasopharyngeal carcinoma, colorectal cancer, gastric cancer, non-small cell lung carcinoma and lung squamous cancer, which is associated with large tumor size and reduced overall survival rate [31,32,33,34,35,36,37,38,39,40]. Conversely, TRIM29 downregulation was detected in prostate cancer, cutaneous head and neck squamous cell carcinoma and breast cancer, in which cancer cell migration and invasion were elevated [41,42,43]. The role of another TRIM family protein, TRIM8, as a ‘double-edged sword’ in promoting or suppressing cancers, was reviewed extensively, illustrating the differential expression of TRIM8 and its respective mode of intervention in regulating cancer-related pathways [44]. Various studies have also suggested the involvement of TRIM proteins in both radio-resistant and chemo-resistant cancer cells [45], for example, TRIM29 and TRIM37, in suppressing the radiosensitivity of lung cancer and colorectal adenocarcinoma [46], and TRIM11, TRIM23, TRIM29 and TRIM65 in promoting the cisplatin-resistant tumorigenesis of various cancers [40,47,48,49].

The disparities in the role of TRIM proteins against malignancies observed may be due to the difference in the physiological nature of cancer cells and cellular pathways where TRIM proteins are involved. TRIM proteins are well known to intervene in cellular pathways through post-translational protein modification, in which the most common way is by proteasomal degradation activity through its RING domain [50]. Intriguingly, TRIM proteins are actively involved in numerous cellular signaling pathways, particularly those that are commonly altered in cancers, including NF-κB, p53, Akt, JAK/STAT, MAPK, TGFβ, Wnt/β-catenin and AMPK [1,11,51,52,53]. Although a recent review has thoroughly elaborated on the upstream regulators and downstream signaling pathways of TRIM proteins in carcinogenesis [11], the role of specific TRIM proteins in mediating the crosstalking of different signaling pathways is not clearly discussed. Thus, this review will focus mainly on the crosstalk of the signaling pathways in cancer development, especially the ones that involve widely studied TRIM proteins, i.e., TRIM28, TRIM25 and TRIM59.

## 2. Key Signaling Pathways in Cancers and Their Signaling Crosstalk: A Brief Overview

### 2.1. NF-κB: Master Switch for Inflammation

The nuclear factor kappa B (NF-κB) family consists of five structurally and functionally related transcription factors, i.e., p65 (RelA), RelB, c-Rel, p105/p50 (NF-κB1) and p100/52 (NF-κB2), where they act as powerful switches in coordinating a wide range of cellular pathways, i.e., from inflammatory responses, apoptosis to cell cycle regulation. The expression of these transcription factors is tightly controlled; when activated, they regulate more than 300 growth- and immune-related genes, including enzymes, cytokines and cell cycle regulators. Numerous human malignancies demonstrate aberrant activation of the NF-κB pathway, which not only aids tumors in evading apoptosis, but also promotes cell cycle progression and increases chemo- and radio-resistance [54,55,56]. NF-κB signaling activation involves the dissociation of NF-κB dimers, releasing inducible NF-κB transcription factors for anti-apoptotic or growth-related genes that support tumor proliferation [57,58]. In quiescent cells, the heterodimers are stabilized by IκBs and localized in the cytosol. Upon ligand binding or stimulation, activated sensing receptors at the cell surface recruit adaptor proteins like TNF-receptor-associated factor (TRAF) family proteins, which later activate IκB kinases (IKKs) for downstream NF-κB activation. During NF-κB activation, activated IKKs phosphorylate and tag IκBs for ubiquitin-mediated proteasomal degradation, thus releasing p65 into the nucleus for transcription.

Several studies have demonstrated the signaling crosstalk of NF-κB with other signaling pathways, such as p53, Akt, JAK/STAT, TGFβ, MAPK and Wnt/β-catenin. Although p53 is an NF-κB-inducible gene, p53 and NF-κB’s p65 mutually repress each other for transcription [59]. In cancer, NF-κB activation is often linked to the inhibition of p53-mediated apoptosis, suggesting the antagonistic role of NF-κB in p53 transcription activity [54]. Crosstalk between NF-κB and p53 signaling was observed through IKKβ-mediated p53 suppression, which promotes melanoma carcinogenesis [60]. The physical interaction and phosphorylation of p53 by IKKβ were demonstrated, leading to p53’s polyubiquitination and degradation [56]. NF-κB responsive sites were also discovered in the Mouse double minute 2 homolog (MDM2) promoter (which is a primary negative regulatory factor of the p53 protein), thus suggesting the negative regulatory role of NF-κB in p53 signaling [61]. In prostate cancer, IKK activity was linked to Akt/mTOR signaling activation, which proposes a probable NF-κB-Akt signaling crosstalk [62,63]. NF-κB activation through the PI3K/Akt axis was also demonstrated in promoting cell proliferation in numerous cancers [57].

Interestingly, NF-κB and STAT3 share an overlapping group of cancer-related genes, indicating the crosstalk between NF-κB and the cancer-promoting JAK/STAT signaling [64]. Both NF-κB and STAT3 are indispensable for cancer development and progression in gastric, liver and colon cancers. Physical interactions were also demonstrated between NF-κB’s p65 and p50 with STAT3. In addition to STAT3, NF-κB also synergistically cooperates with STAT1 for the expression of inflammatory genes, although the mechanisms are still limited [65]. Similarly, TGFβ signaling was also frequently linked with NF-κB activation in various cancers [66]. Physical interactions were demonstrated between Smad3 with NF-κB or with IKKα. In addition, the pivotal role of IKKβ in MAPK activation also suggests the possible crosstalk between the NF-κB and MAPK signaling pathway [63]. Although β-catenin was also found to indirectly support NF-κB signaling through the activation of p38 MAPK and various β-catenin genes, NF-κB signaling was also found to be negatively regulated by Wnt/β-catenin during inflammation. Negative regulatory mechanisms include NF-κB sequestration, IκB upregulation, sensing receptor Toll-like receptor 4 (TLR4) downregulation, NF-κB corepressor recruitment and NF-κB acetylation inhibition [67]. These suggest the complex role of Wnt/β-catenin in cancer.

### 2.2. p53: Guardian of the Genome

The p53 protein, encoded by the TP53 gene, is one of the major tumor-suppressive proteins involved in biological function regulation, ranging from DNA metabolism and senescence to innate immunity. TP53 mutation is present in over half of human malignancies, reflecting its undeniable role in cancer development [68,69]. p53 is a DNA-binding global transcription factor governing more than 500 target genes, of which the p53-binding promoter regions are commonly known as p53 DNA response elements [70,71]. As a tumor suppressor, p53 controls the apoptotic events through the regulation of Bcl-2, death receptor Fas and BH3-domain proteins, or cellular senescence through ras oncogene expression. p53 also mediates cell cycle arrest through interaction with various cyclin-dependent kinase (CDK) and CDK-inhibitors, such as Cdc2 and p21, respectively. The expression p53 is commonly induced upon DNA damage with the help of p53’s DNA binding domain. Mechanistically, the activation of p53 triggers DNA damage repair machinery, arrests cell cycle progression and limits retrotransposon activity, thus safeguarding the genome from genetic instability or the accumulation of mutations [72]. Unlike NF-κB, which has its set of signaling molecules, the modulation of p53 occurs through a variety of post-translational modifications, for instance, ubiquitination, acetylation, methylation and sumoylation [70]. p53 activity is generally self-regulated by a constitutive negative feedback loop through various p53 target genes, such as MDM2, Pirh2, COP1, CARPs, etc. MDM2 is the key negative regulator, through repressing TP53 gene transcription, promoting p53 nuclear export and enhancing the ubiquitination and degradation of p53 [73].

Albeit the critical role of p53 signaling in genome maintenance and the removal of damaged cells, many signaling pathways (especially those involved in carcinogenesis) have demonstrated their crosstalk with p53 signaling. This includes NF-κB (as described previously), Akt, JAK/STAT, TGFβ, MAPK, AMPK, PKC and Wnt/β-catenin pathways. Signaling crosstalk between p53 and the tumor-promoting Akt pathway is frequently observed, particularly through Akt-mediated MDM2 phosphorylation and nuclear export, which subsequently elevates p53 degradation activity [74]. As part of the negative feedback loop mechanism, p53 also suppresses Akt signaling by activating tumor suppressor PTEN, a negative regulator of PI3K/Akt phosphorylation. The activation of Akt/mTOR1 signaling is also associated with a decrease in p53 level and growth arrest during malignancy [75]. A recent review by Goyal et al. [76] also revealed a negative correlation and regulatory role between JAK/STAT and p53 signaling through activated STAT proteins. To elaborate, STAT1 was found to stabilize p53 (from degradation) by decreasing MDM2 expression and accentuating various apoptotic genes. However, through its interaction with p53, both STAT3 and STAT5 negatively regulate p53, while also being deregulated by p53. In addition, the protein inhibitor of activated STAT protein gamma (PIASγ) was also found to inhibit p53 transactivation activity.

TGFβ1 is known to act synergistically with wild-type p53 as a tumor suppressor by activating p53 through phosphorylation and enhancing its interaction with Smads for transcriptions of several genes, such as p21, p15 and PAI-1 [77,78]. The mutation of p53, however, was found to reverse the tumor suppressive effect of TGFβ and support TGFβ-mediated oncogenic miRNA maturation [77]. On the other hand, crosstalk between p53 and MAPK p38 signaling was found to be primarily through p38-mediated p53 phosphorylation or p21 stabilization, while p53-inducible phosphatase Wip1 (or PPM1D) serves as the negative regulatory feedback of MAPK p38 signaling [79]. The modulation of p53 activity and MDM2 expression were also reported through the Ras/Raf/MEK/MAPK signaling axis [76]. On the other hand, AMPK can directly activate p53 under metabolic stress [80]. The signaling crosstalk of p53 with other signaling pathways, such as PKC and Wnt, were also well described in other reviews [81,82,83].

## 3. TRIM Proteins in Signaling Crosstalk

**Figure 1:**
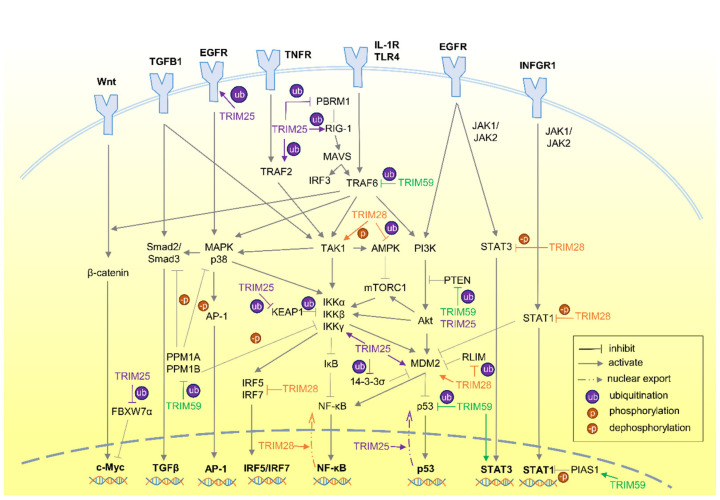
TRIM25, TRIM28 and TRIM59 in the signaling crosstalk between p53, Akt, NF-κB, MAPK p38, TGFβ, AMPK, JAK/STAT and Wnt/β-catenin pathways. For simplicity, only crosstalk that directly involves the interacting protein partners of TRIM25, TRIM28 and TRIM59 are shown in the diagram.

The existing knowledge of signaling pathways involving each TRIM protein merely scratches the surface of the intricate cellular signaling network, especially with increasing studies suggesting that TRIM proteins function through governing multiple signaling pathways for viral immunity, inflammatory response and cancer development [1,84,85]. On top of that, numerous studies demonstrated the direct interaction of TRIM proteins with cellular signaling molecules, or their indirect effect on the expression and protein level of signaling molecules, for instance, NF-κB [51], p53 [45,53], AKT [86] and TGFβ [52]. Thus, a compilation of signaling crosstalk involving each TRIM protein, especially the direct interaction with signaling molecules, is useful in proposing knowledge gaps or even developing targeted cancer therapeutics. Here, the three most studied TRIM proteins in the past decade, with respect to their effect in both carcinogenesis and signaling pathways, will be discussed, namely TRIM25, TRIM28 and TRIM59. A summary of the involvement of the three TRIM proteins in the signaling crosstalk between pathways is depicted in a simplified diagram in Figure 1.

### 3.1. TRIM25

TRIM25, or estrogen-responsive finger protein (Efp), is an E3 ubiquitin and ISG15 ligase that plays a crucial role in cell development, innate immunity and tumorigenesis. It has garnered increasing attention recently in both cancer development and antiviral immunity studies [87,88,89]. TRIM25 was first identified as an estrogen-responsive gene protein, harboring a RING finger domain at its N-terminal, followed by two B boxes domains, a CC domain and a C-terminal PRY-SPRY domain [90]. As an IFN-inducible protein, its aberrant expression resulted in autoimmunity and cancer development. Its upregulation was frequently observed in various cancers, including hormone-sensitive cancers (breast, ovary, endometrium, prostate), epithelial cancers (liver, colon, stomach, lung) and multiple myeloma [1,11,91]. Mechanistically, numerous studies demonstrated the regulation and direct interaction of TRIM25 with diverse signaling molecules, primarily regulating p53, Akt, NF-κB and MAPK pathways, which directly or indirectly contribute to cell proliferation, migration and tumorigenesis (Figure 1).

TRIM25 was found to bind and promote the ubiquitination and degradation of tumor suppressive 14-3-3σ, which is a negative regulator of MDM2 that accelerates its self-ubiquitination and downregulation [92,93]. The phosphorylated peptide sequence ^402^KLP(pT)FG^407^ at the loop region of the TRIM25’s coiled-coil domain was found to be important for its interaction with 14-3-3σ [94]. In a separate study, TRIM25 was co-precipitated with both tumor-suppressor p53 and p53’s inhibitor MDM2, where its binding with MDM2 attenuated p53 polyubiquitination and subsequent degradation [95]. However, p53 transcriptional activity did not increase with the TRIM25-elevated p53 protein level, but was inhibited upon overexpression of TRIM25 [96]. A recent study confirms the oncogenic role of TRIM25, though a different mechanism was demonstrated, with TRIM25 = promoting p53 nuclear export through incorporation with SNORD15B in endometrial cancer cells or the G3BP2 complex, and then nuclear p53 SUMOylation in prostate cancer cells [97,98]. At the upstream of p53 signaling, TRIM25 also mediates the K63-linked ubiquitination of PTEN, a suppressor of Akt/mTOR signaling, thus reducing the nuclear translocation of PTEN and subsequently the activation of Akt/MDM2 p53 degradation [99,100]. Taken together, oncogenic TRIM25 directly binds to p53, MDM2 and PTEN in p53-Akt signaling crosstalk for p53 suppression and Akt activation.

In recent years, several immunology studies have revealed the oncogenic role of TRIM25 through the activation of the NF-κB signaling pathway. A co-immunoprecipitation assay showed direct interaction between TRIM25 and NF-κB upstream activators including IKKα, IKKβ and IKKγ, which are essential for the full activation of MAVS-dependent NF-κB signaling [101]. In addition to NF-κB signaling kinases, the receptor retinoic acid-inducible gene-I (RIG-I) protein has long been known as an interacting partner with TRIM25, in which TRIM25 ubiquitinates and activates it for the IRF3 and NF-κB signaling response [102,103]. On top of the direct activation of RIG-I, TRIM25 also physically binds to and promotes the ubiquitination and degradation of RIG-I signaling repressor chromatin remodeler polybromo-1 (PBRM1), further enhancing its downstream interferon production [104].

The effect of TRIM25 on signaling crosstalk between the MAPK and NF-κB signaling pathway involves the regulation of several TNF-receptor-associated factor (TRAF) family proteins. As the downstream effectors of TNF receptors, TRAFs evidently activate the NF-κB, MAPK and IRF signaling pathways [105]. TRIM25 physically interacts with and promotes the K63-linked polyubiquitination of TRAF2, which is essential for interaction with TAK1 or IKKβ and the activation of TNF-α-induced NF-κB signaling [106]. The activation of NF-κB signaling through TRIM25-dependent TRAF6 ubiquitination was also observed, although no physical binding was detected [101]. Both TRAF2 and TRAF6 were RING domain-containing E3 ubiquitin ligases that were directly involved in the activation of NF-κB and MAPK signaling pathways [107,108]. The activation of TRAF2 and TRAF6 by TRIM25 may concurrently activate both NF-κB and MAPK signaling, which in turn promotes cell proliferation and tumorigenesis. Interestingly, TRIM25 also directly ubiquitinates the Myc inhibitor, FBXW7α, and thus reduces the degradation of oncogenic Myc protein [109].

Physical interaction between TRIM25 and epidermal growth factor receptor (EGFR) was observed in lung cancer, where TRIM25 enhances EGFR expression and increases EGFR stability (by promoting the ubiquitination of the 63rd lysine site of EGFR) for persistent EGFR signaling activation and lung cancer development [110]. The activation of EGFR signaling was linked to various cancer development, such as lung cancer and glioma, through its downstream signaling crosstalk with the MAPK, Akt, STAT3 and PKC signaling pathways [111,112,113].

Just like TRIM40 in esophagus cancer, TRIM25 also binds to E3 ligase adaptor Kelch-like ECH-associated protein 1 (Keap1), and promotes its ubiquitin-mediated degradation and subsequently Nrf2 nuclear translocation and activation [114,115]. The downregulation of Keap1 activates Nrf2 for cell growth and the survival of hepatocellular carcinoma. Increasing studies have demonstrated the involvement of the Keap1-Nrf2 signaling axis in multiple signaling networks including the Akt, NF-κB, MAPK and TGFβ pathways [116].

TRIM25 also interacts with Krüppel-like factor 5 (KLF5). However, TRIM25 did not ubiquitinate KLF5, despite TRIM25 overexpression being solely responsible for the negative regulation of KLF5 [117]. This suggests an alternative pathway of KLF5 degradation by TRIM25, especially when KLF5 is a key downstream transcription factor of multiple signaling pathways, such as TGFβ and Wnt signaling [118]. The expression of TRIM25 was also observed to positively correlate with the Smad2 and Smad4 expression levels, constructing its link to TGFβ signaling pathway, although the underlying mechanisms are yet to be identified [119].

### 3.2. TRIM28

TRIM28, also known as KRAB-associated protein 1 (KAP1) and transcription intermediary factor 1-beta (TIF1β), is a versatile transcriptional regulator mediating cell growth, homeostasis and DNA repair [120,121]. Its expression was commonly associated with poor prognosis and cancer metastasis [120]. TRIM28 demonstrated its pivotal function as a transcriptional co-repressor, especially with the huge transcription factor family Krüppel-Associated Box Zinc Finger Protein (KRAB-ZFP), transcriptionally regulating growth-related and tissue-specific genes [122,123]. Intriguingly, studies also suggested the alternative KRAB-ZFP-independent mechanism of TRIM28 for its function, through RNA polymerase regulation, chromatin relaxation and EMT activation [120,124,125,126]. It is therefore crucial to understand the multiple signaling networks that TRIM28 is involved in. To date, TRIM28 was found to directly participate in the p53, AMPK, NF-κB and JAK/STAT signaling pathways (Figure 1), while the association of its expression with Akt, MAPK, Wnt/β-catenin and TGFβ signaling was also observed.

The co-immunoprecipitation of TRIM28 with MDM2 was demonstrated by Wang et al. [127], where their interaction enhances the ubiquitination and degradation of p53. A recent structural study revealed the binding of TRIM28 to another E3 ubiquitin ligase RLIM, the negative regulator of MDM2 [128]. The regulation of MDM2/p53 signaling was also observed at the upstream of TRIM28. Suppressor Of Cytokine Signaling 1 (SOCS1) interacts directly with TRIM28’s Bromo/PHD domain, possibly sequestering it from stabilizing MDM2, and thus activating p53 signaling in osteosarcoma [129]. On the other hand, in skeletal tissue, Sentrin-specific protease 6 (SENP6) binds with and stabilizes TRIM28 through desumoylation, resulting in p53 suppression [130].

The oncogenic effect of TRIM28 on the MDM2/p53 and AMPK/mTOR signaling pathways was also observed to emerge from its physical interaction with several oncogenic Melanoma Antigen Gene (MAGE) proteins. Aberrant expression of the MAGE protein family was linked to tumorigenesis, in part through interactions with E3 ubiquitin ligases, particularly TRIM28, TRIM31, TRIM69 and TRIM27 [131]. MAGE-A3 was discovered to suppress p53 activity through the direct interaction and upregulation of TRIM28 [132]. In addition, the MAGE-A protein targets the cellular energy homeostasis regulator AMPK for ubiquitination and degradation, thereby upregulating mTORC signaling and suppressing cell autophagy [133,134]. At the downstream of TRIM28, MAGE-C2 was protected from ubiquitin-mediated proteasomal degradation by TRIM28 [135]. TRIM28’s binding with MAGE proteins expands the role of TRIM28 in cancer signaling crosstalk.

TRIM28 knockdown also significantly attenuated NF-κB gene expression and decreased cell proliferation and migration in vascular smooth muscle cells [136]. Consistent with this observation, Liang and co-workers discovered that TRIM28 is required for the K63-linked ubiquitination of Receptor-Interacting Protein Kinase 1 (RIPK1), which is crucial for NF-κB pathway activation [137]. Interestingly, several studies demonstrated the anti-tumor role of TRIM28 through the negative regulation of nuclear NF-κB. TRIM28 sequesters NF-κB from transcriptional activity, which was antagonized by Receptor-Interacting Protein Kinase 3 (RIPK3) binding and phosphorylation, that lead to NF-κB transactivation [138]. The formation of the TRIM28-NF-κB complex also disrupts the binding between NF-κB and p300, inhibits NF-κB’s acetylation and subsequently promotes its nuclear export [139].

Intriguingly, TRIM28 also negatively regulates the JAK/STAT signaling pathway through direct interaction with STAT3 and STAT1. TRIM28 binds to cytoplasmic STAT3, inhibits its phosphorylation from downstream JAK/STAT signaling activation and thus impairs radiation-induced DNA damage repair in bone marrow hematopoietic cells [140]. TRIM28 knockdown elevated STAT3 nuclear translocation and phosphorylated STAT3 levels in the nucleus [140,141]. Recently, overexpression of TRIM28 was shown to promote hematopoiesis through STAT3 inhibition in acute myeloid leukemia [142]. TRIM28 also interacts better with phosphorylation-mutant STAT1 than its wild type, possibly trapping it to regulate JAK/STAT signaling and IRF-1 expression [143,144,145].

The involvement of TRIM28 in multiple signaling pathways was also demonstrated by its interaction with other popular transcription factors and oncogenic proteins. To illustrate, TRIM28 enhances the SUMOylation of IRF7 and suppresses its downstream transcriptional activity [146]. Similarly, TRIM28-IRF5 binding leads to abrogated IRF5-mediated TNF expression [147]. TGFβ-activated kinase 1 (TAK1), a multiple signaling regulator of MAPK, NF-κB, p53, TGFβ, Wnt and AMPK pathways, was also found to interact with and was activated by TRIM28 through enhanced autophosphorylation [148,149,150]. A positive correlation between TRIM28 expression with MAPK and NF-κB signaling (both canonical and non-canonical) was also linked to their upstream activation of TNFR signaling by TRIM28, though the underlying mechanism warrants further structural studies [151]. The interaction of TRIM28 with oncogenic EZH2 was also observed, and is found to be critical for EZH2 protein stability and mammosphere formation in breast cancer [152,153]. EZH2 expression was found to be closely linked with various signaling pathways, including p53, NF-κB, MAPK, EGFR and WNT/β-catenin [154,155,156,157,158,159].

### 3.3. TRIM59

TRIM59, also called RING finger protein 104 or RNF104, is an E3 ubiquitin ligase harboring RING domain, a B-box domain, a CC domain and a putative transmembrane (TM) domain [160]. Oncogenic TRIM59 is often found to be overexpressed and serves as a strong prognostic factor for poor patient survival in multiple cancers [161,162]. Although the TRIM59 studies are comparably less than other popular TRIM proteins, TRIM59 has caught increasing attention over the last five years, with astounding findings, especially its involvement in multiple signaling pathways. TRIM59 demonstrated direct interactions with several signaling proteins involved in p53, Akt, NF-κB, MAPK, TGFβ and JAK/STAT pathway (Figure 1).

The ubiquitination activity of TRIM59 on the p53 and Akt signaling network contributes to its tumor-promoting effect in several cancers. TRIM59 binds directly to the p53 protein and enhances its ubiquitination and subsequent proteasomal degradation [163]. The p53 signaling suppression by TRIM59, however, only reduces the protein level of p53, and not its mRNA level, which is responsible for aberrant cell proliferation and migration in osteosarcoma [164]. TRIM59-dependent suppression of p53 signaling was spotted in liver, gastric, pancreatic and breast cancers [24,163,165,166]. At the upstream of p53, TRIM59 targets PTEN for its ubiquitination and degradation, therefore activating PI3K/Akt/mTOR signaling and further suppressing the downstream p53 signaling pathway through MDM2 [167]. The expression of TRIM59 correlates with elevated Akt, PI3K and mTOR phosphorylation in breast, colorectal, lung, ovarian and pancreatic cancer and cholangiocarcinoma [20,23,165,167,168,169].

TRIM59 is also involved in the signaling crosstalk between JAK/STAT and EGFR pathways through physical interaction between TRIM59 and nuclear STAT3, STAT 1 and Protein Inhibitor of Activated STAT 1 (PIAS1). TRIM59 binds with and inhibits STAT3’s dephosphorylation by T-cell protein tyrosine phosphatase (TC45), resulting in persistent transcriptional activation and gliomagenesis [170]. Another study demonstrated the pivotal role of TRIM59 as an effector of EGFR signaling to activate the STAT3 pathway, which enhanced the gefitinib resistance of lung adenocarcinoma [171]. Both studies also illustrated that upregulation of TRIM59 by EGFR signaling, further strengthens their protein–protein interactions. On the other hand, another protein partner of TRIM59, i.e., PIAS1, an E3 SUMO ligase, was found to promote the dephosphorylation of STAT1. The direct interaction between TRIM59 and PIAS1 significantly enhances PIAS1-STAT1 interaction, thereby suppressing the JAK2-STAT1 signaling pathway and nitric oxide (NO) production in macrophages, which subsequently promotes melanoma growth [172]. This observation is aligned with recent work by Wang and co-workers, who demonstrated that TRIM59 can interact, ubiquitinate and degrade STAT1 [173].

Crosstalk between TRIM59 and NF-κB, p38/MAPK and TGFβ signaling was discovered through direct interaction between TRIM59 and metal-dependent protein phosphatases (PPM), such as PPM1A and PPM1B. PPM1A and PPM1B target IKKβ upstream of NF-κB to synergistically suppress TNFα-mediated NF-κB activation [174,175]. TRIM59 interacts with and enhances PPM1B ubiquitination and degradation, promoting the proliferation and migration of hepatocellular carcinoma through the upregulation of p-IKKβ and downstream NF-κB signaling activation [176]. On the other hand, the ubiquitination activity of TRIM59 on PPM1A leads to PPM1A’s proteasomal degradation, which suppresses the dephosphorylation of the p-Smad2/3 and consequently activates the TGFβ/Smad signaling pathway [177]. PPM1A, which is also known as a master switch, also directly dephosphorylates ERK, p38 and JNK, and subsequently attenuates MAPK signaling pathways [178,179,180].

In addition to PPM, TRIM59 also interacts with the upstream NF-κB signaling activator TRAF6. TRAF6 upregulation is commonly associated with tumor development and metastasis, which is also often linked to the activation of NF-κB, MAPK, Akt, AP-1, Wnt and YAP pathways [181]. In contrast to its common oncogenic role, TRIM59-TRAF6 binding leads to K48-linked ubiquitination and proteasomal degradation of TRAF6, suppressing NF-κB signaling and promoting the BECN1-mediated autophagy pathway in non-small cell lung cancer [182]. The mechanisms behind the dual effect of TRIM59 on the NF-κB pathway, whether it is the upregulation of the NF-κB pathway through PPM or negatively through the TRAF6-mediated pathway, are worth studying.

Several proteins involved in autophagy, inflammation and innate immunity have also demonstrated physical interaction with TRIM59. TRIM59 interacts and stabilizes programmed cell death protein 10 (PDCD10) by sequestering it from ubiquitination by transmembrane domain-containing protein 1 (RNFT1) and subsequent autophagic degradation [22]. PDCD10 overexpression leads to enhanced cell proliferation and migration through the suppression of Rho-associated coiled-coil containing protein kinase (ROCK1)-mediated cell plasticity in breast cancer [22,183]. The TRIM59-mediated ubiquitination and degradation of abhydrolase-domain-containing 5 (ABHD5) promote lung cancer progression through NLRP3 inflammasome signaling activation and IL-1β production [184]. Although the physical binding of TRIM59 to evolutionarily conserved signaling intermediate in Toll pathways (ECSIT) was observed, their interaction did not lead to ECSIT’s ubiquitination, which is pivotal for NF-κB activation [185,186,187]. The oncogenic effect of TRIM59 can also be observed through the Wnt/β-catenin signaling pathway, where TRIM59 overexpression upregulated β-catenin and enhanced cell proliferation in neuroblastoma [188], but whether the effect is inherited from other signaling crosstalk or the direct interaction of TRIM59 with Wnt signaling molecules is still unknown.

## 4. Summary, Limitations and Future Directions

TRIM proteins are aberrantly expressed and correlate with poor prognosis and metastasis in many cancers. TRIM25, TRIM28 and TRIM59 are consistently upregulated in cancers such as colorectal cancer, suggesting their oncogenic function [189,190]. To date, all three studied TRIM proteins were found to directly participate in NF-κB and p53 signaling pathways, while TRIM25 and TRIM59 also demonstrated direct interactions with several signaling proteins in Akt and MAPK pathways. On the other hand, both TRIM28 and TRIM59 were also involved in direct interaction with signaling molecules in JAK/STAT pathways, while TRIM28 and TRIM59 were found to directly participate in AMPK and TGFβ signaling pathways, respectively. These interactions directly or indirectly contribute to cell proliferation, migration and tumorigenesis.

Intriguingly, although all three TRIM proteins were involved in the same NF-κB and p53 signaling pathways, the reported interacting protein partners for the TRIMs are mostly different from each other. For example, in the NF-κB signaling pathway, TRIM28 activates IKKα/β/γ’s upstream regulator, TAK1, while TRIM59 and TRIM25 ubiquitinate negative regulators of IKKβ, i.e., PPM1A/B and KEAP1, respectively. Similarly, in the Akt/p53 pathway, TRIM25 and TRIM28 activate MDM2 via ubiquitination of the negative regulator of MDM2, 14-3-3σ, and RLIM, respectively, while TRIM59 ubiquitinates p53 directly. This is not surprising, as the three TRIM proteins belong to different classes of TRIM with different substrate recognition domains. For instance, TRIM25 has the PRYSPRY domain (Class IV), TRIM28 consists of the PHD-BRD domain (Class VI) and TRIM59 has a putative TM domain (Class XI).

The examples above also clearly demonstrate how the three TRIM proteins can work synergistically in activating both NF-κB and Akt/p53 pathways (though this may not always be the case). Less commonly, some TRIM proteins are also found to share a common interacting partner. For instance, both TRIM25 and TRIM59 are known to directly ubiquitinate PTEN and subsequently activate the Akt pathway. This may suggest a common binding domain between TRIM25 and TRIM59 on PTEN (which may provide an opportunity for the design of a pan TRIM-PTEN inhibitor), though structural information about the interactions may be required first to confirm this.

Each TRIM also has protein partners in various signaling pathways, in agreement with its ubiquitous properties. Interestingly, although all three TRIM proteins are generally oncogenic, some of their interactions with the protein partners have tumor suppressing effects. To illustrate this, TRIM28 binds to cytoplasmic STAT3 and inhibits its phosphorylation from downstream JAK/STAT signaling activation. Similarly, TRIM59 was found to ubiquitinate and negatively regulate TRAF6, and subsequently decrease NF-κB signaling activation. These provide an avenue for the selective targeting of specific protein–protein interactions as potential therapeutics, though this may not be an easy task, considering the complex network of crosstalk between multiple signaling pathways.

Interestingly, the three TRIM proteins were also found to be involved in regulating proteins that act like master switches, mediating crosstalk between multiple signaling pathways. For example, TRIM28 activates TAK1 via phosphorylation, which is involved in activating the MAPK, Wnt, NF-κB and Akt/p53 signaling pathways. Similarly, TRIM25 activates EGFR via ubiquitination, which is linked to the activation of the JAK/STAT, PKC, Akt/p53 and MAPK signaling pathways. For TRIM59, its direct binding partner, PPM1A, is involved in the deactivation of the NF-κB, TGFβ and MAPK signaling pathways. This opens the possibility of preventing the activation of multiple signaling pathways concurrently by just inhibiting a specific protein–protein interaction.

It is, however, important to note that the existing knowledge of signaling pathways involving each TRIM protein (and their protein partners) is still quite limited, though new findings have consistently continued to emerge. Additionally, this review only focuses on three TRIM proteins and a few key signaling pathways involved in cancer development; hence, more information on other signaling pathways involving the three TRIM proteins, such as the Notch1 signaling pathway, or involving other TRIM family proteins should be sought elsewhere [191,192]. The interaction between the TRIM proteins and their upstream regulators, including the host single-stranded RNA or the double-stranded RNA, in oncovirus in cancer cells is also a worthy topic to be further explored, as this type of interaction has been found to also affect TRIM protein ubiquitination activity, as well as its subcellular localization, which may ultimately affect its overall functionality [193].

## Data Availability

Not applicable.
